# The Effect of Anesthesia Method on Melatonin Level and Its Relationship With Postpartum Depression ın Cesarean Section Patients

**DOI:** 10.7759/cureus.70344

**Published:** 2024-09-27

**Authors:** Remziye Ayşenur Nalbant, Ufuk Kuyrukluyildiz, Didem Onk, Sara Salcan, Nurcan Kutluer Karaca, Taha Abdulkadir Çoban, Suheyla Unver, Nurhan Eren

**Affiliations:** 1 Department of Anesthesiology and Reanimation, Erzincan Binali Yıldırım University, Erzincan, TUR; 2 Department of Epidemiology and Public Health, Erzincan Binali Yıldırım University, Erzincan, TUR; 3 Anesthesiology and Reanimation, Faculty of Medicine, Erzincan Binali Yıldırım University, Erzincan, TUR; 4 Department of Biochemıstry, Erzincan Binali Yıldırım University, Erzincan, TUR; 5 Department of Anesthesiology and Reanimation, University of Health Sciences, Ankara Oncology Training and Research Hospital, Ankara, TUR

**Keywords:** cesarean section, general anesthesia, melatonin, postpartum depression, regional anesthesia

## Abstract

Objective

Our study aimed to establish the effects of anesthetic agents used in cesarean sections on melatonin levels and to evaluate the possible association between melatonin levels and postpartum depression.

Materials and methods

Our study was approved by the Erzincan Binali Yıldırım University Ethics Committee (approval number: 25.07.2017 11/11). We included 231 pregnant women between the ages of 18 and 45 who were admitted for a cesarean section to Erzincan Mengücek Gazi Training and Research Hospital Anesthesiology and Reanimation Clinic. The pregnant women were divided into three groups that underwent general, spinal, and combined spinal-epidural anesthesia, respectively. These approaches were determined by the preference of the attending anesthesiologist. On the postoperative first day at 5:00 am, peripheral venous blood samples were obtained from the subjects for melatonin level measurement. Samples were centrifuged and kept in -80 °C until the testing for melatonin with the enzyme-linked immunosorbent assay (ELISA) method. In order to determine the depression status, the subjects were evaluated with the Edinburgh Postpartum Depression Scale.

Results

Of all the anesthesia types given to the subjects, 16% was combined spinal-epidural anesthesia, 18.2% was general anesthesia, and 65.8% was spinal anesthesia. According to the scale, 68% of the subjects had no depression and 32% had depression. The depression rate in subjects who underwent general anesthesia was significantly higher than in other groups (p<0.001). The association between the anesthesia method and melatonin levels had no statistically significant difference (p=0.53). The association between depression status and melatonin levels had no statistically significant difference among the two groups (p=0.097).

Conclusion

We aimed to evaluate the effect of the chosen anesthesia method on postoperative melatonin levels and postpartum depression and found that the chosen method of anesthesia does not affect postoperative melatonin levels significantly but the usage of general anesthesia significantly raises the postpartum depression rate among subjects. We also did not find any correlation between postoperative melatonin levels and postpartum depression.

## Introduction

Due to a good number of reasons like older age of first pregnancy, fear of delivery room and labor pain, increase in assisted reproductive methods and multiple pregnancies, and women's decision about the mode of delivery days beforehand, there is an increase in cesarean section delivery rates nowadays [1].

Hormonal, behavioral, and psychological aspects of living organisms consist of a rhythm, often called the circadian rhythm. The impairment, delay, or advancement of the circadian rhythm is closely linked to numerous diseases and mood disorders [2]. The best measurement of circadian rhythm is the melatonin hormone [3].

Melatonin is recognized as a strong antioxidant hormone, which is mainly produced by the pineal gland, especially in dark environmental settings. A disruption in the circadian rhythm is commonly observed in numerous clinical disorders such as mood disorders [2]. There are many studies evaluating the effect of melatonin on depression in the literature. Our aim in this study is to determine the effect of anesthesia methods used in cesarean section on melatonin levels and to investigate its relationship with postpartum depression.

## Materials and methods

Following the Ethics Board approval of Erzincan Binali Yıldırım University (EBYU)Faculty of Medicine (Approval Number: 25.07.2017 11/11), this cross-sectional study was conducted with the contributions of the EBYU Scientific Research Projects Coordination Unit (Project Code: TTU-2018-590) at the Erzincan Mengücek Gazi Teaching and Research Hospital Anesthesiology and Reanimation Clinic. The sample size for our study was calculated as 195 at a 95% confidence level, with 80% power, given that the postpartum depression rate of the reference study was taken as 15% [4].

A total of 243 pregnant women between the ages of 18 and 45 who will give birth via a cesarean section were included in our study. Patients who didn’t accept to participate in our study, patients under the age of 18 and over the age of 45, patients who smoke, have mental retardation, have psychiatric illnesses, use anti-psychotics, anti-depressants, have sleep disorders, receive hormone replacement therapy, have difficulty in understanding and speaking were excluded from our study.

The women were informed about the study protocol and procedures, and written consents were obtained for each individual. Each subject was monitored in the operating room beforehand and 2-4 lt/min oxygen was administered with a face mask for pre-oxygenation. The method of anesthesia for each subject was determined by different anesthetists in the operating room. Parameters such as the anesthesia method (general, spinal, and combined spinal-epidural), age, number of pregnancies, and type of operation (elective, emergency) were recorded retrospectively from the anesthesia form. According to the anesthesia technique applied, patients were divided into three groups: general, spinal, and combined spinal-epidural.

Blood samples were taken from each subject through the peripheral venous access at 05:00 am on the postoperative first day to measure blood melatonin levels. The samples were centrifuged and then stored at -80 degrees Celsius. Blood samples were studied with the BioTek 800 TS Microplate Absorbance Reader device (Agilent Technologies, Santa Clara, California, US) via the use of the enzyme-linked immunosorbent assay (ELISA) method and melatonin hormone kits.

The Edinburgh Postpartum Depression Scale was used on the postpartum seventh day to measure depression.

Statistical analysis

The collected data were evaluated by the SPSS for Windows 22.0 (IBM Corp. Released 2013. IBM SPSS Statistics for Windows, Version 22.0. Armonk, NY: IBM Corp.) statistical software package. According to the Shapiro-Wilk normal distribution test, which is used in quantitative variables. Edinburgh Depression Scale scores didn’t match the normal distribution of postoperative melatonin levels (p<0.05). As a result, the numerical variables were described as median and minimum & maximum values; and the categorical variables were described as number (n) and percentage (%) in our analysis. Mann-Whitney U and Kruskal Wallis tests were used for the variables that didn’t show normal distribution, and Pearson chi-square analysis was used for the categorical variables. The Spearman correlation test was used to evaluate the relationship between continuous variables. A p-value of <0.05 was considered statistically significant.

## Results

A total of 243 pregnant women who gave birth by cesarean section were included in our study. Twelve pregnant women who met the exclusion criteria were taken off our study.

The age average of the 231 included women was 30.31 ± 5.57 in our study. Among all the subjects, 63.6% (n=147) of the women had two or fewer pregnancies beforehand and 36.4% (n=84) of the women had three or more. According to the type of procedure, 48.1% (n=111) of the women underwent elective and 51.9% (n=120) underwent emergency operations, respectively (Table 1).

**Table 1:** Distribution of various characteristics of pregnant women *Data in the table are presented with percentages (n (%)) for categorical variables.

	n	%
Age Groups (n/%)		
25 and below	44	19.1
26-35	147	63.6
36 and above	40	17.3
Number of Pregnancies (n/%)		
1	58	25.1
2	89	38.5
3 and more	84	36.4
Operation Timing/Type (n/%)		
Elective	111	48.1
Emergency	120	51.9

When the types of anesthesia techniques were evaluated, 16% (n=37) of combined spinal epidural anesthesia, 18.2% (n=42) of general anesthesia, and 65.8% (n=152) of spinal anesthesia were observed (Table 2).

**Table 2:** Distribution of anesthesia techniques among pregnant women *Data in the table are presented with percentages (n (%)) for categorical variables. CSEA: combined spinal epidural anesthesia

	n	%
Anesthesia Type (n/%)		
General	42	18.2
CSEA	37	16.0
Spinal	152	65.8

When the depression status of the pregnant women was evaluated, it was found that 68% (n=157) of the women did not have depression while 32% (n=74) had depression (Table 3).

**Table 3:** Distribution of depression status among pregnant women *Data in the table are presented with percentages (n (%)) for categorical variables.

Depression status (n/%)	n	%
No	157	68.0
Yes	74	32.0

**Figure 1:**
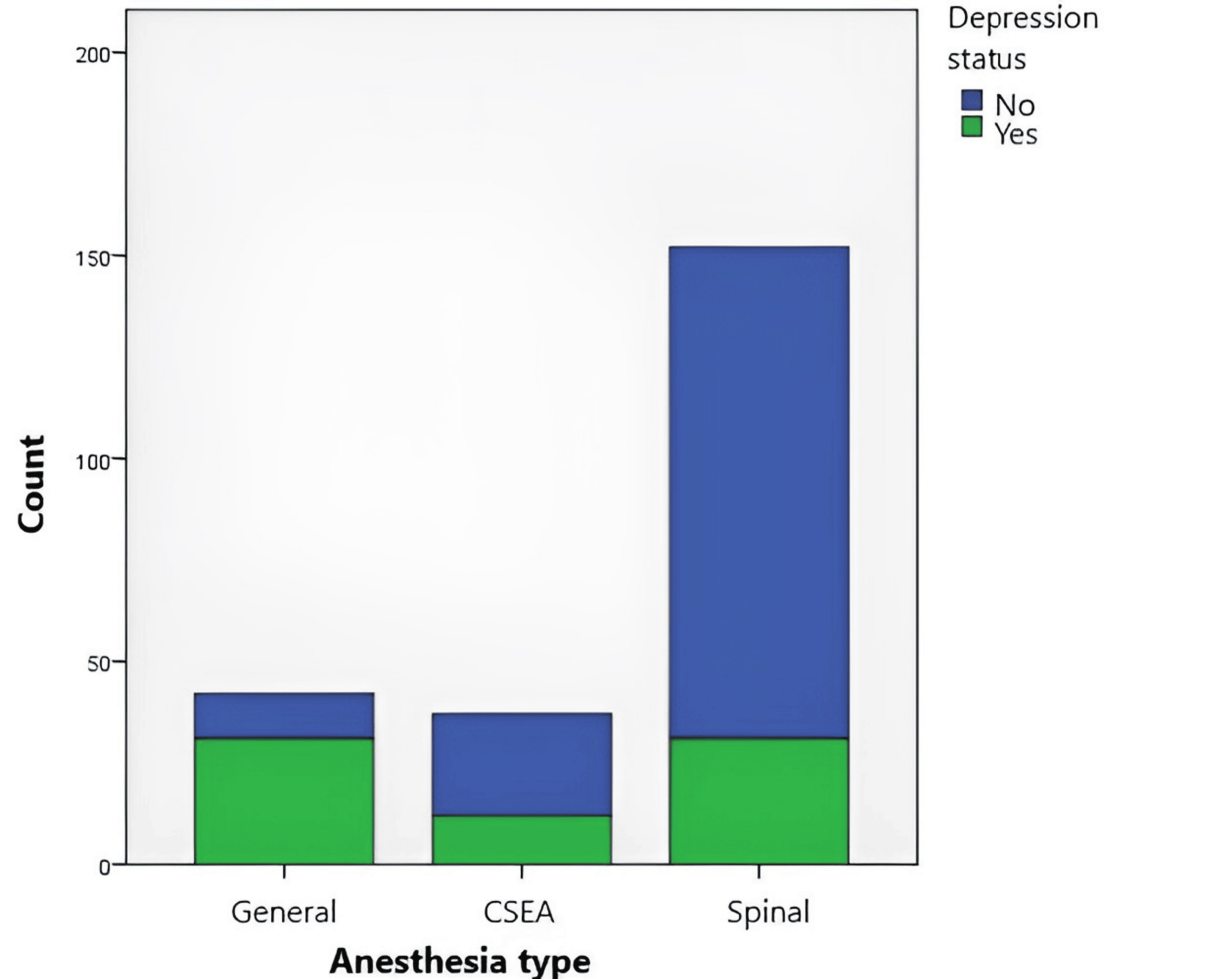
Comparison of the depression scale scores of pregnant women, according to the method of anesthesia *Pearson chi-square analysis was used for the categorical variables. **p <0.05 is considered significant. CSEA: combined spinal epidural anesthesia

When the Edinburgh Post-Depression Rating Scale (EPDRS) scores were evaluated according to the anesthesia method, the median score of pregnant women who underwent general anesthesia was 13.5, the median score of those who underwent CSEA was 11, and the median score of those who underwent spinal anesthesia was 8; hence, a significant difference was found between groups (p<0.001) (Figure 1).

Among the women who participated in our study, 32.0% had postpartum depression. When the depression status was evaluated according to the anesthesia methods used, depression was observed in 31 (73.8%) women who received general anesthesia, 12 (32.4%) women who received CSEA, and 31 (20.4%) women who underwent spinal anesthesia, respectively. The depression rate in women who underwent general anesthesia was found to be significantly higher than in other groups (p<0.001) (Table 4).

**Table 4:** Comparison of depression status in pregnant women according to the anesthesia method * Data in the table are presented with percentages n (%) for categorical variables; ** Pearson chi-square analysis was used for the categorical variables; *** p <0.05 is considered significant CSEA: combined spinal epidural anesthesia

	Depression Status					
	No		Yes		p	Chi-Square Value
Anesthesia Method	n	%	n	%	< 0.001*	43.126
General	11	26.2	31	73.8		
CSEA	25	77.6	12	32.4		
Spinal	121	79.6	31	20.4		

**Figure 2:**
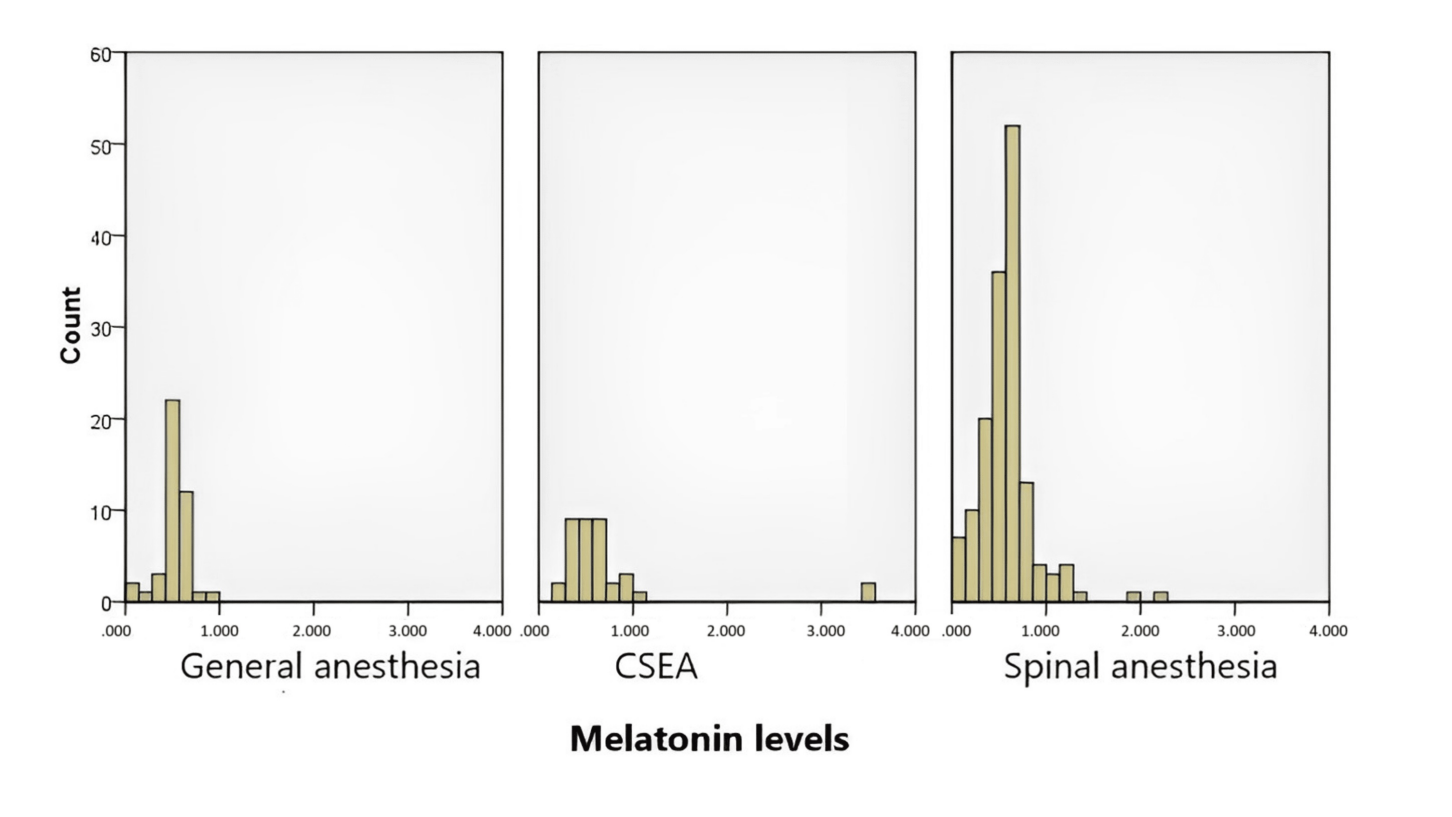
Comparison of melatonin levels in pregnant women according to the anesthesia method * Kruskal Wallis tests were used for the variables, which didn’t show normal distribution; ** The data in the table are presented as median (min-max) values for continuous variables; *** p <0.05 is considered significant. CSEA: combined spinal epidural anesthesia

When the relationship between melatonin levels was compared according to the anesthesia method, the median melatonin levels for patients who received general anesthesia, CSEA, and spinal anesthesia were 0.54, 0.56, and 0.58, respectively. The difference between melatonin levels according to the used method of anesthesia was not statistically significant (p=0.53) (Figure 2).

When the relationship between depression status and melatonin levels among women was examined, it was found that the median melatonin level of women without depression was 0.55, and the median melatonin level of those with depression was 0.59; hence, no significant difference was found between groups (p=0.097) (Table 5).

**Table 5:** Comparison of melatonin levels in pregnant women according to the anesthesia method * The Mann-Whitney U test was used for the variables, which didn’t show normal distribution; ** The data in the table are presented as median (min-max) values for continuous variables; *** p <0.05 is considered significant.

Depression Status	Melatonin Levels: med (min-max)	p	z
No	0.55 (0.04-3.53)	0.097 *	-1.658
Yes	0.59 (0.05-1.90)		

**Figure 3:**
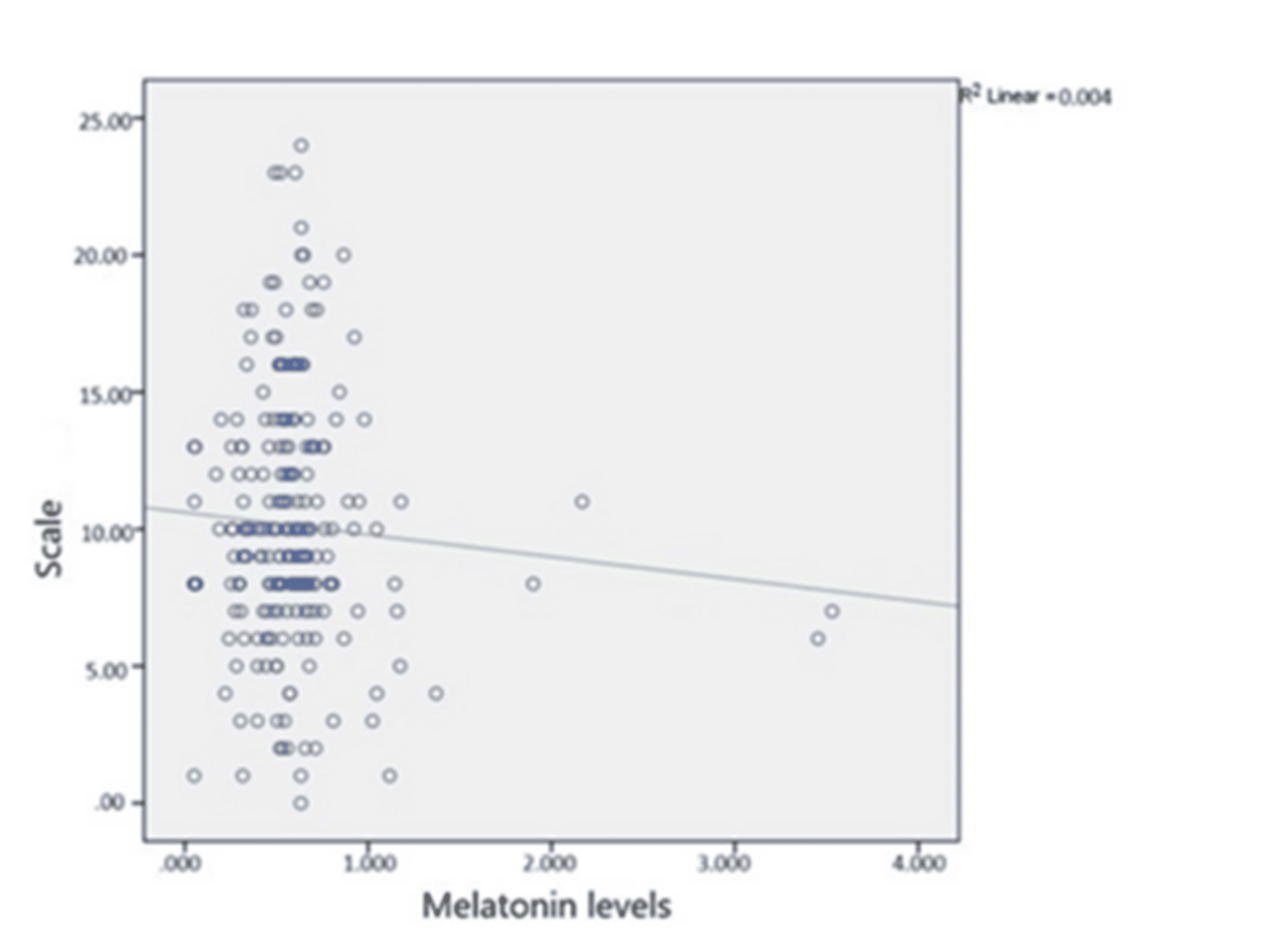
Correlation between melatonin levels and EPDRS scores among pregnant women * r: Correlation factor; if the correlation coefficient is between r = 0-0.39, it indicates a weak correlation; if it is between r = 0.40 and 0.69, it indicates a moderate correlation; and if it is r = 0.70 and above, it indicates a high correlation; ** p <0.05 is considered significant; *** The Spearman correlation test was used to evaluate the relationship between continuous variables.

When the correlation between melatonin levels and the Edinburgh Post-Depression Rating Scale (EPDRS) scores of all women was evaluated, no significant relationship was found (r= -0.14; p>0.05) (Figure 3).

## Discussion

In this study, we evaluated the effect of anesthesia methods on postoperative melatonin levels and its relationship with postpartum depression in cesarean section patients. Our findings suggest that the anesthesia methods did not alter the postoperative melatonin levels significantly, but the rate of postpartum depression was significantly higher in pregnant women who had general anesthesia. Additionally, our results did not indicate any relation between postoperative melatonin levels and postpartum depression.

Although for the health of the mother and baby, it is crucial to select a suitable anesthesia method with the cesarean section; the decision of the method is based upon the urgency of the surgery, clinical status of the patient, experience of the anesthesiologist, and preference of the patient.

There is increasing evidence suggesting a substantial increase in regional anesthesia methods being used in our country [5,6]. In a study conducted by Aksoy et al., it was observed that the rate of regional anesthesia increased from 34% in 2003 to 69% in 2012. When the most commonly used regional anesthesia methods were evaluated, even though only spinal anesthesia was used in 2003, they found that 41% of the regional anesthesia methods were spinal anesthesia and 27% were combined spinal-epidural anesthesia in 2012 [7]. In our study among 231 women, 65.8% of them underwent spinal anesthesia, 37% underwent CSEA, and 42% underwent general anesthesia.

Psychiatric disorders may occur during pregnancy and postpartum period in pregnant women with psychosocial sensitivity, and genetic or biological predisposition. In a study conducted by Xie et al. including 415 patients, postpartum depression was detected in 21% of women who delivered by cesarean section and 10.9% of women who delivered vaginally [8]. In another meta-analysis, cesarean delivery was found to be associated with postpartum depression [9].

When we evaluated all pregnant women in our study, we found no postpartum depression among them. When we scrutinized the question "Does the anesthesia method have an effect on postpartum depression?", we observed that the rate of postpartum depression was significantly higher in women who had cesarean section with general anesthesia (p<0.05). We believe that general anesthesia is a risk factor in addition to many risk factors that cause postpartum depression in pregnant women. In recent years, cesarean delivery has become increasingly popular due to better pain management with regional anesthesia and the reduction of prejudices in society [10]. We believe that postpartum depression is less common in women who deliver with spinal-epidural anesthesia due to the long duration of postoperative analgesia, the mother's ability to see her baby right after birth, and the ability to breastfeed briefly after [7].

The analgesic property of melatonin in its wide spectrum of actions is among its most substantial effects. Experimental studies have shown that melatonin has strong dose-dependent analgesic effects [11]. Even though some evidence suggests that melatonin increases the release of β-endorphins from the pituitary gland, it is believed that naloxone may antagonize melatonin-induced nociceptive effects by blocking the binding of β-endorphin to opioid receptors [12]. The fact that long-term analgesia caused by melatonin can be blocked via naloxone suggests that melatonin affects the opioid receptors [13].

In a study conducted by Kihezri et al., 120 women who underwent spinal anesthesia during cesarean section were randomly divided into three groups. Twenty minutes before spinal anesthesia, 3 mg of anesthesia was administered to the first group, 6 mg to the second group, and a sublingual placebo to the third group [14]. It was found that the total analgesic requirement of the patients during the postoperative 24 hours was significantly lower in the group that was given 3 mg of melatonin as compared to the placebo group, and there was no significant difference between the 6 mg of melatonin and the placebo group.

It is a well-known fact that pain-related anxiety can increase the intensity of the pain perceived [15]. Pain and anxiety are often the consequence of surgery. Therefore, pain and anxiety associated with environmental factors like hospitalization, medication, stress, and general anesthesia can affect the sleep and wake cycle of patients. Safe and effective analgesia is a necessity in women with cesarean section. A good analgesia increases mobilization and may reduce the risk of thromboembolism, which is increased during pregnancy [16,17]. Pain may also negatively affect the mother's ability to give optimal care for her baby in the postpartum period.

Plasma melatonin levels can fluctuate in the postoperative period and in hospitalized patients [18]. When the anxiety rates of Kihezri et al.'s study were evaluated, it was found that the anxiety rate of the group given 6 mg of melatonin was significantly lower than other groups. In another study conducted by Patel et al. with 120 cases; anxiety rates were found to be significantly lower in the melatonin group compared to the placebo group [19]. We found that the chosen anesthesia methods in our study did not cause a significant difference in the melatonin levels, although melatonin levels were lower in women who underwent general anesthesia compared to other methods.

In a study conducted by Yamak et al., among pregnant women who delivered by elective cesarean section, 30 were randomly selected, and CSEA was applied to the first group and general anesthesia to the second group. It was found that the need for additional analgesia was significantly higher in the general anesthesia group [20]. We think that melatonin might be a viable option for the treatment of postoperative pain in pregnant women who go under general anesthesia. In addition, we believe that postoperative pain is also a reason for the high depression rate among the women who underwent general anesthesia in our study.

One of the limitations of our study is that we have conducted numerous spinal anesthesias due to reasons such as pregnant women specifying the anesthesia method they want us to use beforehand, obstetricians preferring this method due to its many advantages over regional anesthesia, and the experience of the anesthesiologist.

When we summarize the studies conducted, we determine that melatonin has many positive effects on pregnant women, just like every other human being. However, we could not find any study examining the effect of anesthesia methods on melatonin levels when we searched the current literature. Our study is the first in the literature, to the best of our knowledge, to examine the effects of anesthesia methods on melatonin hormone levels and the relationship between melatonin hormone levels and postpartum depression. We hope that our study will guide new studies with much greater sample sizes in the future.

## Conclusions

In conclusion, our study found that anesthesia methods did not have a significant effect on melatonin levels, although the depression rate was significantly higher in the postpartum period in pregnant women who received general anesthesia.
